# Editorial: Glyphosate Herbicide as Endocrine Disruptor and Probable Human Carcinogen: Current Knowledge and Future Direction

**DOI:** 10.3389/fendo.2021.772911

**Published:** 2021-10-04

**Authors:** Virginia Lorenz, María Florencia Rossetti, Eliane Dallegrave, María Mercedes Milesi, Jorgelina Varayoud

**Affiliations:** ^1^ Instituto de Salud y Ambiente del Litoral (ISAL), Facultad de Bioquímica y Ciencias Biológicas, Universidad Nacional del Litoral (UNL) - Consejo Nacional de Investigaciones Científicas y Técnicas (CONICET), Santa Fe, Argentina; ^2^ Departamento de Bioquímica Clínica y Cuantitativa, Facultad de Bioquímica y Ciencias Biológicas, Universidad Nacional del Litoral (UNL), Santa Fe, Argentina; ^3^ Pharmacosciences Department, Federal University of Health Sciences of Porto Alegre, Porto Alegre, Rio Grande do Sul, Brazil; ^4^ Cátedra de Fisiología Humana, Facultad de Bioquímica y Ciencias Biológicas, Universidad Nacional del Litoral (UNL), Santa Fe, Argentina

**Keywords:** glyphosate, endocrine disruptor, adverse reproductive effects, multigenerational effects, thyroid function, epigenetic modifications

Glyphosate-based herbicides (GBHs) are the pesticides most globally used and the rate of usage does not appear to slow. Although farmers, applicators and their families are the most susceptible population, some biomonitoring studies are indicating that glyphosate and aminomethylphosphonic acid (AMPA), its main metabolite, are found in biological fluids in populations from non-agricultural contexts (1, 2). This is a real concern because the extent of the population potentially exposed to glyphosate herbicide is greater than we think and the consequences become more difficult to restrain.

In the current scenario, this Research Topic aims to revise, analyze and compile the state of the art of the two most controversial issues about the herbicide glyphosate: its potential as an endocrine-disrupting compound and as a probable human carcinogen. The present Research Topic includes different kinds of articles: an original article (Gorga et al.), two reviews (Milesi et al. and Romano et al.) and three mini-reviews (Anderson et al., Rodríguez et al. and Rossetti et al.). These articles describe different models of studies and possible targets of action of glyphosate herbicide which are closely related with female and male reproduction, reproductive outcomes, hormonal balance and the epigenome addressed by specialists in their fields.

In this sense, the mini-review by Anderson et al. focuses on the endocrine-disrupting effects of technical-grade glyphosate and GBHs on sex hormone pathways with impact on the reproductive system of male and female mammals. Moreover, authors discuss potential sources of controversies which might explain why the position of United States Environmental Protection Agency (3) and the European Food Safety Authority (EFSA) (4) do not correlate with several past and latest findings supporting the disrupting behavior of this herbicide. Regarding male reproductive toxicity of glyphosate, the article by Gorga et al. provides original data about the effects of low doses of glyphosate and a GBH formulation on blood-testis barrier function in juvenile rats showing this is a sensitive endpoint. According to Gorga et al., the impairment detected at the prepuberal stage might be a reversible phenomenon since sperm production was not affected in adult animals. A meta-analysis which covered studies using doses in the order of magnitude or higher than Gorga et al. has suggested that exposure to the herbicide causes decrease in sperm concentration in rodents (mice and rats) (5). We think it would be of great interest to know whether populations of young men exposed to continuous and low doses of the herbicide could suffer alterations in their reproductive capabilities in adult life. Meanwhile, the review by Milesi et al. focuses on female fertility. First, Milesi et al. give an overview of the main routes and current levels of human exposure to GBHs in order to put readers in context. Then, researchers analyze evidence about glyphosate and GBHs as potential estrogenic compounds. Finally, they address the effects of glyphosate and GBHs on female reproductive outcomes and the detrimental effects reported in the successive generations (multigenerational effects) in animal models with particular emphasis on maternal exposure.

On the other hand, an interesting and less studied area includes the effects of glyphosate and GBHs on hypothalamus–pituitary–thyroid (HPT) axis which is addressed in the review by Romano et al. Authors hypothesize whether glyphosate and/or GBHs could be associated with a higher incidence of thyroid disorders. They describe the regulation of HPT axis and thyroid hormone balance highlighting potential susceptible points for the herbicide, and analyze evidence regarding the repercussions of glyphosate herbicide on HPT axis. Importantly, recent works indicate that the gastrointestinal tract play an important role in the control of thyroid function and apparently, GBH can disrupt intestinal microbiome in different models of study (Romano et al.). Therefore, studies associated with gut-thyroid axis will be relevant in advancing the knowledge about the effects of glyphosate herbicide on thyroid function.

While previous articles focus their research in mammalian models, Rodríguez et al. propose an original approach to study reproductive effects with an estuarine crab species relevant for trophic chain: *Neohelice granulate.* The mini-review by Rodríguez et al. summarizes *in vitro* and *in vivo* evidence about the effects of glyphosate and GBHs on ovarian maturation and sperm production in crabs, which suggests that glyphosate herbicide could be an endocrine disruptor.

A novel mechanistic insight about glyphosate herbicide is discussed in the mini-review by Rossetti et al. where authors summarize the current evidence about epigenetic modifications induced by glyphosate, GBHs and AMPA, both in human cells and rodents. Rossetti et al. propose that epigenetic changes could be possible mediators by which these chemicals alter physiological processes in a transient or permanent way along generations. Interestingly, authors suggest that new studies in that direction would contribute to know how epigenetic markers are dysregulated in human disease and to recognize windows of vulnerability by herbicide exposure.

Concerning the potential of glyphosate as a probable carcinogen, although the International Agency for Research on Cancer (6) from the World Health Organization established glyphosate as a probable human carcinogen, EFSA concluded that the herbicide does not prove to be carcinogenic or mutagenic (7). These controversies push our laboratory group and others to investigate and increase knowledge about this issue using *in vitro* and *in vivo* approaches (8–10). Due to evidence published in epidemiological studies is not conclusive to indicate that glyphosate is a compound that denotes a risk for developing cancer in humans (11), some research groups are evaluating associations between the presence of glyphosate in biological fluids and certain types of cancer (12).

The main aspects about glyphosate and GBHs that emerges from this Research Topic are: i) the importance of studying very carefully the implications of co-formulants in pesticide mixtures in order to approve their usage and not only evaluate the active ingredient. The case of GBHs is an interesting example due to GBH formulations available in the market are countless and their inert ingredients have not been explored in depth or considered in the evaluations, ii) further epidemiological evidence is a priority to evaluate the potential adverse effects of glyphosate and GBHs on human populations and particularly, on the most sensitive ones (pregnant women and children).

Finally, in the near future some initiatives could be implemented until new epidemiological studies regarding GBHs and pesticides in general come to light. To the extent that governments authorize the use of certain pesticides and genetic modified crops, we think that they should be compromised surveillance agents with a watchful eye in the levels of residues of agrochemicals in foods and inert matrixes such as water and air. For instance, it should be encouraged farmers to deliver their production with the minimum levels of pesticides. The state should put in high priority the control of the levels of the agrochemicals most applied on fruits, vegetables, pulse, seeds and raw materials for the production of processed foods, often being the basis of differentiated diets (for instance vegetarian or vegan diets), as a preventive measure of diseases or future pathologies. Another issue that closely touches Argentina for being one of the countries with the highest consumption of agrochemicals, as well as Brazil, USA, among others is the quality of the air we breathe. In this sense, some researchers from the National Institute of Agricultural Technology (INTA) in Argentina have started biomonitoring the air of certain periurban areas and are developing devices in order to do that (13).

## Author Contributions

All authors contributed to the article and approved the submitted version.

## Conflict of Interest

The authors declare that the research was conducted in the absence of any commercial or financial relationships that could be construed as a potential conflict of interest.

## Publisher’s Note

All claims expressed in this article are solely those of the authors and do not necessarily represent those of their affiliated organizations, or those of the publisher, the editors and the reviewers. Any product that may be evaluated in this article, or claim that may be made by its manufacturer, is not guaranteed or endorsed by the publisher.

## Abbreviations

AMPA, aminomethylphosphonic acid; EFSA, European Food Safety Authority; GBHs, glyphosate-based herbicides; HPT, hypothalamus–pituitary–thyroid.
